# Ambient air pollution, weather changes, and outpatient visits for allergic conjunctivitis: A retrospective registry study

**DOI:** 10.1038/srep23858

**Published:** 2016-04-01

**Authors:** Jiaxu Hong, Taoling Zhong, Huili Li, Jianming Xu, Xiaofang Ye, Zhe Mu, Yi Lu, Alireza Mashaghi, Ying Zhou, Mengxi Tan, Qiyuan Li, Xinghuai Sun, Zuguo Liu, Jianjiang Xu

**Affiliations:** 1 Department of Ophthalmology and Visual Science, Eye, and ENT Hospital, Shanghai Medical College, Fudan University, 83 Road Fenyang, Shanghai, China; 2 Eye Institute of Xiamen University, Road Xiang’an Nan, Xiamen, China; 3 Shanghai Key Laboratory of Meteorology and Health, 951 Road Jinxiu, Shanghai, China; 4 Massachusetts Eye and Ear Infirmary, Harvard Medical School, Boston, Massachusetts 02114, USA; 5 State Key Laboratory of Medical Neurobiology, Institutes of Brain Science, Fudan University, Shanghai, China; 6 Myopia Key Laboratory of the Ministry of Health of China, Shanghai, China; 7 Department of Environmental Science, Westminster College, Fulton, MO 65251, USA.

## Abstract

Allergic conjunctivitis is a common problem that significantly impairs patients’ quality of life. Whether air pollution serves as a risk factor for the development of allergic conjunctivitis remains elusive. In this paper, we assess the relationship between air pollutants and weather conditions with outpatient visits for allergic conjunctivitis. By using a time-series analysis based on the largest dataset ever assembled to date, we found that the number of outpatient visits for allergic conjunctivitis was significantly correlated with the levels of NO_2_, O_3_, and temperature, while its association with humidity was statistically marginal. No associations between PM_10_, PM_2.5_, SO_2_, or wind velocity and outpatient visits were seen. Subgroup analyses showed that sex seemed to modify the effects of humidity on outpatient visits for allergic conjunctivitis, but not for NO_2_, O_3_, or temperature. People younger than 40 were found to be susceptible to changes of all four parameters, while those older than 40 were only consistently affected by NO_2_ levels. Our findings revealed that higher levels of ambient NO_2_, O_3_, and temperature increase the chances of outpatient visits for allergic conjunctivitis. Ambient air pollution and weather changes may contribute to the worsening of allergic conjunctivitis.

With the rapid industrialization and urbanization, air quality problems and other environmental health troubles are particularly becoming major sources of morbidity and mortality in human^1^. Allergic conjunctivitis is one of the most common ocular surface diseases; it damages the ocular surface and causes debilitating symptoms of redness and itching, resulting in impaired quality of life for patients and limited physical activity^2^. More importantly, allergic conjunctivitis affects up to 40 percent of the population of the United States^3^.

Pollen, animal dander, and other environmental antigens are the main causes of allergic conjunctivitis, yet a recent (2014) hospital-based study showed that exposure to ambient air pollution—especially particulate air pollution—possibly increases the risk of allergic conjunctivitis^4^. The effects of exposure to other well-defined pollutants such as ozone (O_3_), sulfur dioxide (SO_2_), and nitrogen dioxide (NO_2_) on this disease remain inconclusive. Although several studies have evaluated this issue, with limited success^4^,^5^, we still lack a long-term study with a large sample size to make statistically significant statements. Furthermore, human health is being adversely affected by climate change^6^. This change in weather patterns has been proven to affect the timing, distribution, quantity, and quality of aeroallergens, and has changed the distribution and severity of asthma and allergic disease^7^; its role in the development of allergic conjunctivitis, however, remains unknown.

The effect of ambient air pollution and weather pattern changes on allergic conjunctivitis may differ from asthma and other allergic diseases due to anatomical differences between the eyes and lungs. For example, unlike the lungs, where air pollutants tend to penetrate deeply and stay, the cleansing system of the ocular surface (i.e. tears) may be capable of alleviating the cellular damage induced by air pollutants. On the other hand, the ocular surface is directly exposed to the environment, which means that it may be more susceptible to weather changes.

Taken together, we hypothesize that ambient air pollution as well as weather changes, are both associated with risk for allergic conjunctivitis. To address this issue, population-wide data is crucial. The current study exemplifies a unique situation whereby population-wide outpatient attendance for allergic conjunctivitis in Shanghai was recorded systemically from 2008 to 2012, as well as the information on ambient air pollutants and weather conditions. The availability of such a large dataset allowed us to assess the effects of ambient air pollutants and weather conditions on the prevalence of allergic conjunctivitis with statistical rigor.

## Results

**Figure 1:**
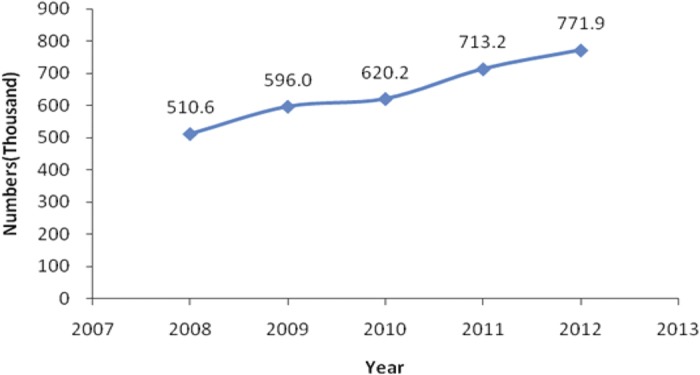
The numbers of outpatient visits with allergic conjunctivitis from 2008 to 2012.

**Figure 2:**
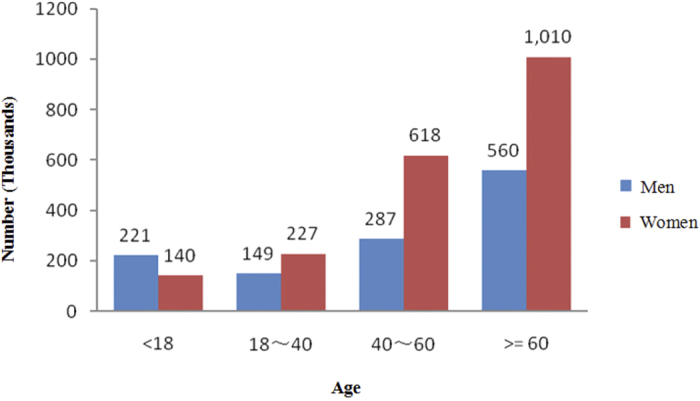
Age distribution of numbers of outpatients with allergic conjunctivitis.

**Figure 3:**
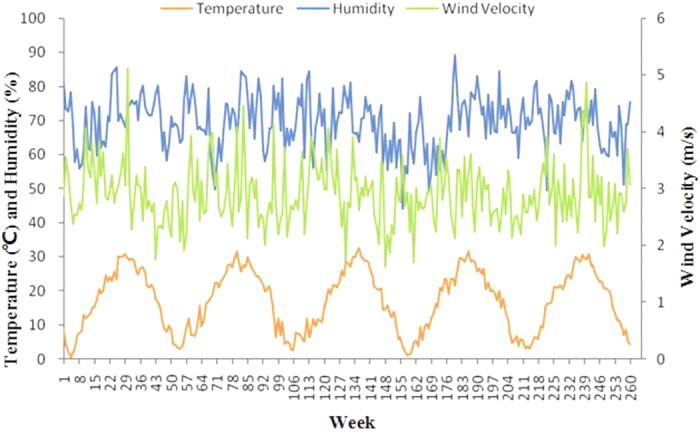
Changes of weather parameters from 2008 to 2012.

**Figure 4:**
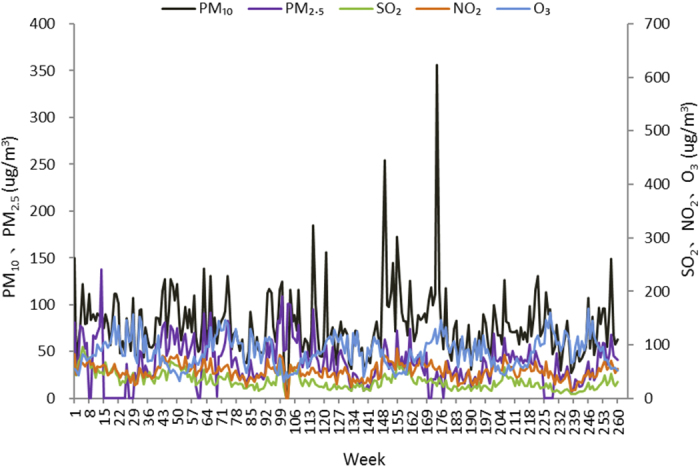
Changes in air pollutants from 2008 to 2012.

The current study included 3,211,820 outpatient visits by 15,938,870 subjects enrolled in SHIS for allergic conjunctivitis. Table 1 and Fig. 1 show the baseline characteristics and the number of outpatient visits for the five-year period. More than three million outpatient visits occurred during the time period examined. The mean age of the patients’ onset was 54 years, and 62 percent were women; the number of outpatient visits was highest in people older than 60 years (Fig. 2). Table 2 lists the concentration of air pollutants and weather conditions, while Figs 3 and 4 further demonstrate the changes in these parameters with time.

**Figure 5:**
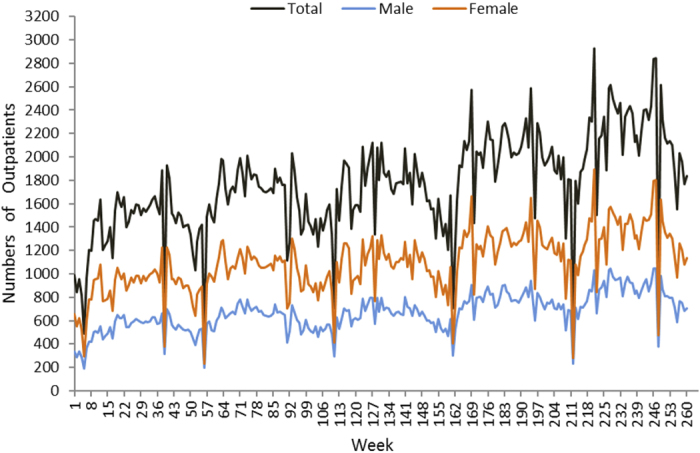
Changes of outpatients with allergic conjunctivitis from 2008 to 2012.

We decided to convert the outpatient visits from a daily to a weekly measure, as we noticed that the outpatient visits showed strong periodicity by week due to the working hours of the local population. After the conversion, the average weekly outpatient visits ranged from 423 to 2,927 per week (Fig. 5). Although the outpatient visits may be mostly described as a Poisson event, the large mean of the weekly visit can be approximated by a Gaussian distribution (Supplemental file 1).

The outpatient visits for allergic conjunctivitis were significantly correlated with the levels of NO_2_, O_3_, and temperature (Table 3). Among the pollutants we evaluated, every 10 μg/m^3^ increased exposure to NO_2_ resulted in 61 more outpatient visits per week, whereas 10 μg/m^3^ higher O_3_ exposure increased the weekly outpatient visits by 21 per week. As for the weather conditions, temperature was the only factor that was significantly associated with outpatient visits. The association between outpatient visits and humidity was statistically marginal. In spite of these factors, our results show no significant association for PM_10_ (particles smaller than 10 μm in aerodynamic diameter), PM_2.5_ (particles smaller than 2.5 μm in aerodynamic diameter), SO_2_, or wind velocity.

**Figure 6:**
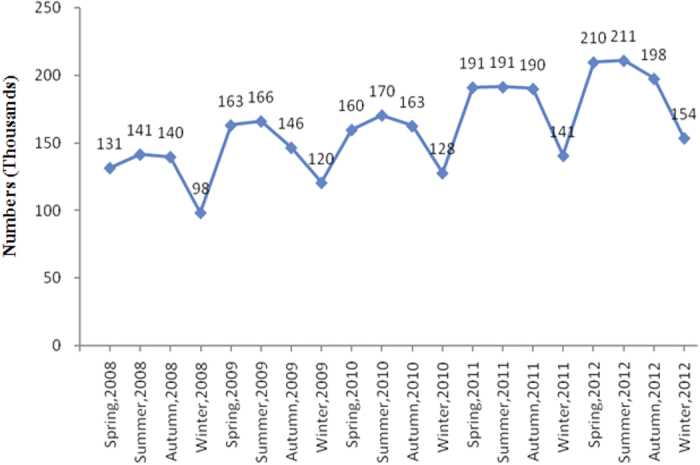
Seasonal changes in the number of outpatients with allergic conjunctivitis.

In order to further investigate the interactions among the pollutants and weather conditions, we tested the interactions between each pollutant and the weather condition, and filtered the interactive factors using the stepwise method (Supplemental file 2). As a result, we found significant interactive effects from NO_2_ and humidity, which suggests that changes in the weather may also affect outpatient visits. Table 4 shows that a one-unit increment in exposure to NO_2_, O_3_, temperature, and humidity elevated the number of outpatient visits from different subgroups. Subgroup analyses showed that humidity—but not NO_2_, O_3_, or temperature—influenced the number of outpatient visits for allergic conjunctivitis, in a sex-dependent manner. People younger than 40 years showed susceptibility to changes in all four parameters, while those older than 40 were only consistently correlated with NO_2_. When the data were stratified according to season, the number of outpatient visits for allergic conjunctivitis was more frequent in the spring and summer than in the winter (Fig. 6 and Table 5). Sex and age did not modify this effect.

## Discussion

During the earliest period of China’s economic reforms, lasting from the late 1970 s to 1985, automobiles were scarce and energy consumption was quite low, which greatly limited emissions of ambient air pollutants and greenhouse gases^8^. From 1985 to 2008, however, the Chinese economy expanded rapidly. Although economic growth associated with industrialization has improved health and various quality of life indicators, environmental issues associated with this process are of increasing concern to the country’s citizens and its government. Our study has demonstrated the relationship between allergic conjunctivitis and air pollution and weather changes. We showed that the number of outpatient visits with allergic conjunctivitis increased as the levels of NO_2_, O_3_, temperature, and humidity changed.

Currently, there are controversies around the association between respiratory allergic disorders and outdoor NO_2_ levels. Hwang *et al*. found that long-term exposure to outdoor NO_2_ increased the subjects’ risks of persistent cough and phlegm, as well as “current asthma” (i.e. people who confirm that they have been told by a health practitioner that they have asthma, and who confirm that they still have it)^9^,^10^. Another earlier study, however, reported that the risk of childhood asthma was not associated with levels of NO_2_ among 32,672 Taiwanese schoolchildren^11^. Information on the association of air pollution with the prevalence of allergic conjunctivitis is limited. Chang *et al*. reported that the air pollutants NO_2_, SO_2_, O_3_, and fine particulate matter can increase the chances of outpatient visits for nonspecific conjunctivitis^5^. Riediker *et al*. found that the rhinoconjunctival tissue is sensitive to irritant stimuli during an ongoing allergic inflammation, and that symptoms of allergic rhinoconjunctivitis might be exacerbated in areas with increased levels of air pollutants^12^. A Japanese hospital-based study also showed that outpatient visits for allergic conjunctivitis had a positive association with air pollutants during a three-month investigation period, although such associations were not statistically significant^4^. Interestingly, among several air pollutants investigated in our study, NO_2_ and O_3_ were the only potent triggers for allergic conjunctivitis exacerbation.

The mechanisms underlying the effects of air pollutants on allergic conjunctivitis are not well understood. The study by Lin *et al*. showed that PM_10_ significantly affects the respiratory system^13^. In the present study, however, no association was found for PM_10_ with allergic conjunctivitis. We assume that PM_10_ and PM_2.5_ do not cause a change in pH (as do other aerosol pollutants after they reach the ocular surface) because they can be cleared out of the ocular surface by the cleansing system of tears^14^, whereas in the respiratory system, fine particulate matter penetrates deeply and stays in the lungs. Furthermore, changes in the lacrimal pH—caused by the acidification of tears exposed to a high-oxidant concentration (NO_2_ and SO_2_)—could irritate the ocular surface^15^,^16^. NO_2_ and O_3_ pose high oxidative potential, and are able to cause damage to the human nasal mucosa^17^; the effects are also apparent in ocular mucosa^18^,^19^. NO_2_ and O_3_ thus may have a higher capacity to provoke conjunctival inflammation than SO_2_ via direct oxidative damage to the ocular surface and acidification of the tears in patients with allergic conjunctivitis. In addition, diesel exhaust pollutants, including NO_2_ and O_3_, have been reported to enhance the allergic sensitization^20^. Furthermore, NO_2_ and O_3_ may induce conjunctival inflammation indirectly via chemical modifications of aeroallergens and subsequent enhanced allergic response.

Human health will likely be adversely affected by accelerating changes in weather patterns^7^. Particularly, allergic diseases may be induced and worsened by weather conditions, because of changes in the type, quantity, distribution, and exposure time of pollens, and because of the interaction between pollens and air pollutants^7^. In the present study, the frequency of outpatient attendance of allergic conjunctivitis was inversely correlated with the humidity, and positively correlated with temperature. The correlation between temperature change and pollen distribution has been well described in the literature. Theoretically, increased temperature stimulates earlier flowering and longer pollen seasons^2^. Results have indicated that if temperature increases under a doubled greenhouse gases scenario by the end of the twenty-first century, pollination seasons will start on average one month earlier, and airborne pollen concentrations will be 50 percent higher than they are today^21^. Meanwhile, low humidity increases land surface evaporation, thus resulting in an increase in airborne pollen concentrations^22^. Pollen sensitivities were indicated as the most frequent triggers for allergic conjunctivitis^23^. Mimura *et al*. reported that Cedar pollen-specific IgE were significantly higher in tears of allergic conjunctivitis patients than in control subjects^24^. These weather pattern changes, in combination with air pollution, will further add to the burden of allergic disease in exposed populations. For patients with allergic conjunctivitis, the results of our study mean that both air quality and weather conditions play important roles in minimizing exposure and symptoms.

Our study also reported the time-lag effect of air pollutants and weather pattern changes on outpatient visits for allergic conjunctivitis. The finding that outpatient visits reached their highest level after three weeks of exposure to pollutants might be of assistance to the public health systems, so that they may better monitor and prepare for environmental events. In addition, the selective and delayed response in patients implies a certain regulatory mechanism that is still not fully understood.

In summary, our study found that higher levels of ambient NO_2_, O_3_, and temperature and lower humidity lead to an increased chance of outpatient visits for allergic conjunctivitis. Our study indicates that ocular surface health may be impaired by long-term exposure to air pollution. These results suggest that efforts to control emissions of both air pollutants and greenhouse gases are warranted. The relationship of these environmental factors to ocular surface health underscores the importance of having all members of the health care team—including health policy decision-makers, specialists, and primary care providers—send the same message to all patients about the necessity of controlling the exposure of the ocular surface to air pollution and adverse weather conditions, as this should help to prevent the onset of allergic conjunctivitis, and should minimize the symptoms and signs in patients who already suffer from the condition.

### Strengths and limitations of the study

While our findings are strengthened by the use of a representative, large-population, registry sample, the present study does have several limitations. First, data on pollen and microbial exposure are not available in our study, which are important outdoor environmental factors associated with allergic conjunctivitis^25^. One may suspect that the overlap time between the concentration of air pollutants and allergens, such as pollens, could bias our findings. Our data, however, did not support this possibility. For example, NO_2_ concentration peaks in winter while pollens are more in spring and summer (Fig. 4). Our findings showed that NO_2_ and O_3_ are independent risk factors that add to the risk posed by the pollens. Second, one may raise the concern about using ambient air pollutant concentrations as exposure surrogates, because people spend more time indoors. However, measurements of indoor air pollutants have a large inter-subject variability. In addition, it seems that the infiltration of ambient pollutants to indoor environments occurs under a high ambient fine particle pollution condition^26^. Real-time ventilation measurements for indoor air pollution are recommended to address this issue. Third, as a registry study, we were not able to take the geographic mobility of the population into account during the follow-up period. Fourth, the SHIS is not solely designated for evaluating the prevalence or clinical characteristics of allergic conjunctivitis. As a result, body mass indexes, sociodemographic situations, education levels, and systemic health conditions were not available for further analysis. This is an inherent limitation of studies that use a pre-existing database. Finally, although we could use the patient’s specific diagnostic codes, uncertainties were unavoidable because the exact diagnosis of each case could not be confirmed without individuals’ medical records.

## Methods

### Allergic Conjunctivitis Data

Data on outpatient visits between January 1, 2008 and December 31, 2012 were obtained from the database of the Shanghai Health Insurance System (SHIS). More than 96 percent of Shanghai’s residents—15.9 million—receive SHIS’s compulsory universal health insurance. All hospitals in Shanghai are required to contract with the SHIS and submit standard claim documents for medical expenses on a computerized form that includes the date of visit and discharge, identification number, sex, birthday, and the diagnosis for each patient. Outpatient visits for allergic conjunctivitis were selected in the current study according to the diagnosis codes of the International Classification of Diseases, ninth revision (ICD-9). The following codes were included: 372.05 (acute atopic conjunctivitis), 372.13 (vernal conjunctivitis), and 372.14 (other chronic allergic conjunctivitis). Only outpatient visits with the aforementioned ICD-9 codes as the major diagnosis were included in the study.

### Air Quality and Weather Data

Daily ambient air-quality data between 2008 and 2012 were obtained from the database of the Shanghai Key Laboratory of Meteorology and Health (SKLMH)^27^. Ambient air pollutants included in the analysis were PM_10_, PM_2.5_, NO_2_, O_3_, and SO_2_. Weather data (daily mean temperature, relative humidity, and wind velocity) were also obtained. All data were collected on an hourly basis. We calculated the daily average for each variable, and then calculated the weekly average for use in this study. The study protocol was approved by the ethics committee of the Shanghai Eye, Ear, Nose, and Throat Hospital and the SKLMH. The requirement for informed consent was waived.

### Statistical Analysis

The univariate generalized least squares (GLS) model was used to evaluate the effect of the environmental factors on the outpatient attendance of allergic conjunctivitis. For the environmental and clinical data, we first took the average by each calendar week to eliminate the periodic trends caused by patients’ regular working hours. The time series of interest were then fit to a simple linear regression model. We applied the Ljung–Box method to the residual of the linear model to determine the correlation structure between the two time series (moving average, autocorrelation) and the corresponding latency (p, q). The Ljung–Box test is to test^28^:

H_0_: The data are independently distributed.

H_a_: The data are dependent on each other, they exhibit serial correlation.

Finally, a GLS model with correlation structure of autoregressive–moving-average (ARMA; p, q) was used to evaluate the association between environmental factors and outpatient attendance. ARMA models are widely used in hydrology^29^, econometrics^30^, and other fields^31^. The ARMA model consists of two parts, an autoregressive (AR) part, represented by *p* (the order of the autoregressive part) and a moving average (MA) part, represented by *q* (the order of the moving average part).

The GLS model is written as:


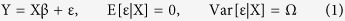


where Y is defined as the outpatient attendance of allergic conjunctivitis, X denotes an environmental factor, ε is the residual error (from which we constructed the ARMA [p, q]), Ω is a known matrix of the conditional variance of the error when given X, and β is an unknown coefficient to be estimated. We called the test *P* of the regression coefficient significant if it was less than 0.05. We used R software version 3.1 (www.r-project.org) for all of the statistical analyses. The R packages we used in this study include “nlme”, “zoo”, and “car”. All *P* values were based on two-sided tests.

In order to determine the time-lag effects of the pollutants, we used the Ljung–Box test to evaluate the significance of correlation between outpatient visits and the concentration of pollutants in the past with time-lags of 1 week to 5 weeks, respectively (Supplemental file 3). The time-lag was then determined as the first time point where the test *P* value became insignificant (more than 0.1). Most of the pollutants were found to show significant time-lag effects in week 3, therefore we established the ARMA [p = 3, q = 0] except for SO_2_, which had a time-lag of 1 week. To set our age groups, we compared the results of principal component analysis (<20, 20–50, and >50 years, Supplemental file 4) with the Chinese hospital standard.for age classification (childhood and adolescence, <18 years; adult, 18 to 40 years; middle age, 41 to 60 years; and elderly, >60 years, Table 4) and found that the two classifications did not match. To facilitate the understanding for clinicians and to have a relatively high resolution, we finally decided to use the latter age classification^32^.

## Additional Information

**How to cite this article**: Hong, J. *et al*. Ambient air pollution, weather changes, and outpatient visits for allergic conjunctivitis: A retrospective registry study. *Sci. Rep.* **6**, 23858; doi: 10.1038/srep23858 (2016).

## Supplementary Material

Supplementary Information

## Figures and Tables

**Table 1:** Descriptive Statistic of the Outpatients of Allergic Conjunctivitis and the Percentages in Gender and Age Group.

Year	Numbers of Outpatient Attendance	Age	Male:Female	Percentage of Patients Younger than 18 Years (%)	Percentage of Patients between 18 and 40 Years (%)	Percentage of Patients between 41 and 60 Years (%)	Percentage of Patients Older than 60 Years (%)
Total	3,211,820	54 ± 23	38%:62%	11.3	11.7	28.1	48.9
2008	510,589	54 ± 22	37%:63%	10.0	12.1	32.1	45.8
2009	596,001	54 ± 23	38%:62%	11.0	11.3	29.6	48.1
2010	620,197	55 ± 23	38%:62%	11.8	10.8	27.7	49.7
2011	713,160	55 ± 23	38%:62%	11.7	11.3	26.6	50.4
2012	771,873	54 ± 23	39%:61%	11.6	12.9	26.1	49.4

**Table 2:** Descriptive Statistic of Air Pollution and Climate in Shanghai by Years and by Seasons between January 2008 and December 2012 (Mean ± SD).

Time	SO_2_ (μg/m^3^)	NO_2_(μg/m^3^)	O_3_ (μg/m^3^)	PM_10_ (μg/m^3^)	PM_2.5_ (μg/m^3^)	Temperature (°C)	Humidity (%)	Wind Velocity (m/s)
Years								
Total	33.2 ± 21.6	51.0 ± 20.9	86.4 ± 37.8	78.4 ± 53.9	40.8 ± 28.2	17.2 ± 9.2	69.4 ± 12.7	2.9 ± 1.0
2008	51.4 ± 26.9	56.3 ± 22.2	85.8 ± 37.8	84.3 ± 45.7	53.3 ± 32.8	17.3 ± 9.2	70.2 ± 12.8	3.0 ± 1.0
2009	33.3 ± 18.1	51.9 ± 19.6	87.2 ± 39.1	77.7 ± 43.6	48.1 ± 31.1	17.5 ± 9.0	70.2 ± 12.6	2.9 ± 1.0
2010	29.2 ± 17.4	49.6 ± 21.7	82.3 ± 37.1	78.7 ± 65.0	38 ± 27.3	17.3 ± 9.1	68.6 ± 12.8	2.9 ± 1.0
2011	29.1 ± 17.9	51.2 ± 20.8	84.2 ± 37.2	79.7 ± 67.4	32.7 ± 22.2	17.0 ± 9.4	68.7 ± 13.0	2.8 ± 0.9
2012	22.7 ± 13.7	46.2 ± 19.0	92.4 ± 37.1	71.3 ± 40.9	35.5 ± 22.4	16.9 ± 9.2	69.6 ± 12.3	2.9 ± 0.9
Seasons								
Spring	33.6 ± 18.9	54.6 ± 18.2	103.3 ± 34.6	89.4 ± 67.4	45.3 ± 28.7	15.7 ± 5.6	65.5 ± 15.3	3.1 ± 0.9
Summer	23.3 ± 13.7	38.2 ± 16.6	99.4 ± 41.7	61.2 ± 33.5	28.5 ± 18.6	27.6 ± 3.1	74.3 ± 8.6	3.0 ± 1.0
Autumn	29.3 ± 17.8	51.9 ± 21.8	85.6 ± 33.0	76.7 ± 52.0	38.7 ± 29.1	19.5 ± 5.5	69.7 ± 10.9	2.6 ± 0.8
Winter	47.1 ± 27.0	59.7 ± 20.5	56.5 ± 18.0	86.5 ± 52.6	51.7 ± 29.5	5.6 ± 3.8	68.1 ± 13.3	2.7 ± 0.9

**Table 3:** Effect of Air Pollutants and Weather Changes on the Number of Outpatient Visits for Allergic Conjuctivits.

Variables	Estimate (95% CI)	*P* value
SO_2_	0.7552 (−0.8182, 2.3287)	0.3460
NO_2_	6.0924 (3.3189, 8.8658)	<0.001
PM_10_	0.5061 (−0.5364, 1.5487)	0.3400
PM_2.5_	0.9368 (−1.0511, 2.9246)	0.3543
O_3_	2.0905 (0.4912, 3.6898)	0.0106
Temperature	18.1008 (7.7422, 28.4594)	<0.001
Humidity	−4.1086 (−8.4040, 0.1869)	0.0607
Wind Velocity	−17.8723 (−78.0138, 42.2691)	0.5589

**Table 4:** Estimated Effect of Change in Variables on Allergic Conjunctivitis Outpatient Visits in Different Groups.

Variables	NO_2_ (per 1 ug/m3)		O_3_ (per 1 ug/m3)		Temperature (per 1 degree)		Humidity (per 1%)	
	Estimate (95% CI)	P value	Estimate (95% CI)	P value	Estimate (95% CI)	P value	Estimate (95% CI)	P value
Total	6.0924 (3.3189, 8.8658)	<0.001	2.0905 (0.4912, 3.6898)	0.0106	18.1008 (7.7422, 28.4594)	<0.001	−4.1086 (−8.4040, 0.1869)	0.0607
Sex								
Male	2.1544 (1.2139, 3.0950)	<0.001	0.8863 (0.3477, 1.4249)	0.0014	7.3758 (3.6772, 11.0744)	<0.001	−1.5614 (−3.0121, −0.1108)	0.0350
Female	3.8846 (2.0280, 5.7412)	<0.001	1.2284 (0.1590, 2.2978)	0.0245	10.6889 (4.1300, 17.2478)	0.0015	−2.5265 (−5.4085, 0.3555)	0.0855
Age at Visit								
<18 years	0.6033 (0.3047, 0.9019)	<0.001	0.5152 (0.3485, 0.6818)	<0.001	3.7876 (2.3842, 5.1910)	<0.001	−0.6813 (−1.1358, −0.2269)	0.0034
19–40 years	0.5337 (0.2448, 0.8228)	<0.001	0.4309 (0.2744, 0.5875)	<0.001	2.5684 (1.3636, 3.7732)	<0.001	−0.6041 (−1.0384, −0.1699)	0.0066
41–60 years	1.5538 (0.8357, 2.2719)	<0.001	0.61205 (0.2053, 1.0188)	0.0033	5.01196 (2.9782, 7.0457)	<0.001	−0.9326 (−2.0511, 0.1859)	0.1018
>60 years	3.2390 (1.5500, 4.9280)	<0.001	0.4736 (−0.5068, 1.4541)	0.3424	6.0473 (−0.1270, 12.2215)	0.0549	−1.6125 (−4.2465, 1.0215)	0.2291

**Table 5:** *ANOVA* Analysis and Multiple Comparisons of Allergic Conjunctivitis Outpatients in Different Seasons.

Variables	Anova Analysis	Spr:Sum	Spr:Aut	Spr:Win	Sum:Aut	Sum:Win	Aut:Win
	*F*(*P* value)	*t*(*P* value)	*t*(*P* value)	*t*(*P* value)	*t*(*P* value)	*t* (*P* value)	*t* (*P* value)
Total	35.4700 (<0.001)	−0.9850 (0.3249)	0.3666 (0.7140)	8.2980 (<0.001)	1.3742 (0.1697)	9.6487 (<0.001)	8.0095 (<0.001)
Sex							
Male	48.3771 (<0.001)	−1.5779 (0.1149)	1.1075 (0.2684)	9.5867 (<0.001)	2.7403 (0.0063)	11.4068 (<0.001)	8.6798 (<0.001)
Female	29.2354 (<0.001)	−0.6592 (0.5100)	−0.0129 (0.9897)	7.5344 (<0.001)	0.6502 (0.5157)	8.6294 (<0.001)	7.6032 (<0.001)
Age at Visit							
<18 years	158.7170 (<0.001)	−7.7357 (<0.001)	2.0497 (0.0407)	13.3466 (<0.001)	10.4818 (<0.001)	23.1597 (<0.001)	12.1149 (<0.001)
19–40 years	119.1145 (<0.001)	−7.4489 (<0.001)	−6.3976 (<0.001)	9.7404 (<0.001)	0.3345 (0.7381)	18.1940 (<0.001)	15.8248 (<0.001)
41–60 years	30.7152 (<0.001)	−0.6433 (0.5202)	0.0823 (0.9344)	7.8772 (<0.001)	0.7147 (0.4750)	8.9061 (<0.001)	7.6086 (<0.001)
>60 years	14.2867 (<0.001)	1.0823 (0.2794)	1.1027 (0.2705)	6.0122 (<0.001)	0.0641 (0.9489)	5.3168 (<0.001)	5.0111 (<0.001)

Spr = Spring; Sum = Summer; Aut = Autumn; Win = Winter.
