# Supplementary material for: Ambient air pollution, weather changes, and outpatient visits for allergic conjunctivitis: A retrospective registry study
Source: Sci Rep. 2016 Apr 1;6:23858. doi: 10.1038/srep23858 (PMC4817244; doi:10.1038/srep23858)
Supplement: Supplementary Information [file srep23858-s1.pdf]

Supplementary Information

Ambient air pollution, weather changes, and outpatient visits for allergic conjunctivitis: A retrospective registry study

Jiaxu Hong, Taoling Zhong, Huili Li, Jianming Xu, Xiaofang Ye, Zhe Mu, Yi Lu, Alireza Mashaghi, Ying Zhou, Mengxi Tan, Qiyuan Li, Xinghuai Sun, Zuguo Liu, Jianjiang Xu

Supplementary File 1: Distribution of weekly outpatient visits for allergic conjunctivitis

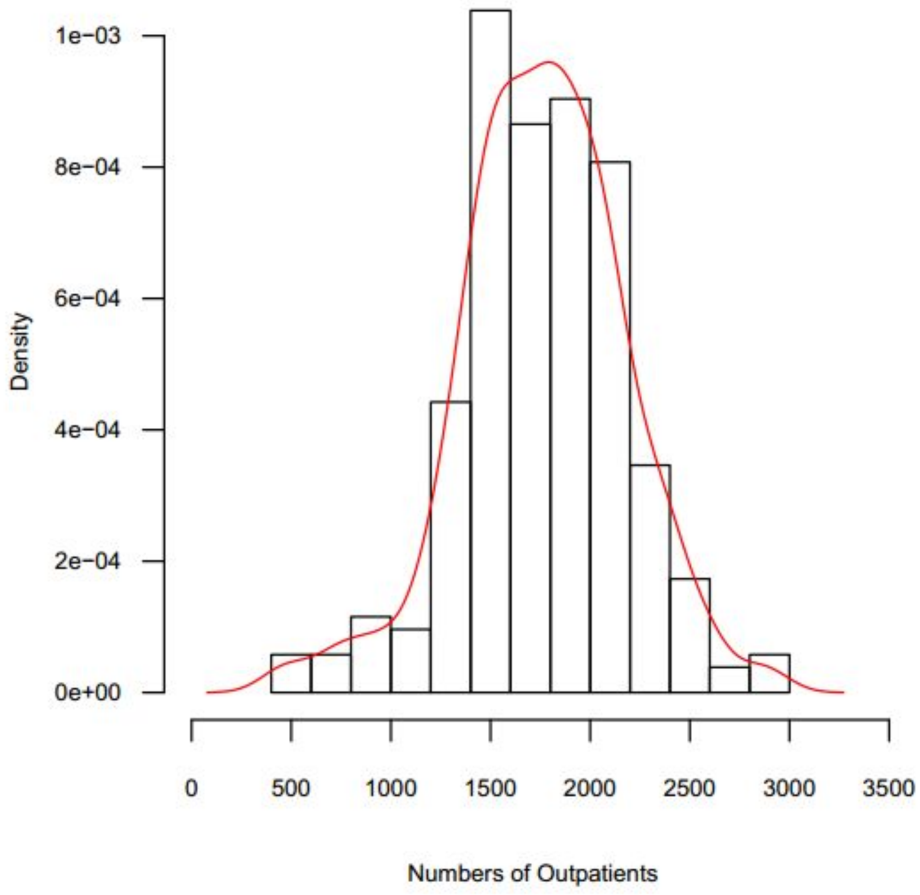

Figure Legend: The Anderson–Darling normality test indicated that the distribution is close to normal distribution ($P = 0.0939$).

Supplementary File 2 Correlation between the Number of Outpatients, with the Interaction between each Pollutant and the Weather Condition by the Stepwise Regression Method

Variables	Estimate (95% CI)	*P* value
**SO_2**	12.942 (-80.66, 106.543)	0.7856
**SO_2: Wind Velocity**	-0.461 (-35.698, 34.776)	0.9795
**NO_2**	267.814 (75.457, 460.172)	0.0065
**NO_2: Humidity**	-2.999 (-5.765, -0.234)	0.0336
**PM_{10}**	-2.555 (-14.654, 9.545)	0.6779
**O_3**	24.411 (-24.092, 72.914)	0.3226
**O_3: Temperature**	-0.431 (-2.339, 1.477)	0.6569

Supplementary File 3 The *P*-value of Ljung-Box Test for the ARMA Model to Fit the Residual of the Univariate Linear Model when Latency is from 1 to 5 Weeks

Variables	lag=1	lag=2	lag=3	lag=4	lag=5
**SO_2**	0.4501	0.5845	0.9562	0.9901	0.9708
**$N0_2$**	0.0074	0.0245	0.6414	0.9442	0.8835
**PM_{10}**	0.0008	0.0094	0.5096	0.8696	0.8338
**O_3**	0.0051	0.0082	0.4706	0.7899	0.4054
**Temperature**	0.0026	0.0046	0.3423	0.7650	0.4852
**Humidity**	0.0001	0.0019	0.4564	0.9421	0.8453
**Wind Velocity**	0.0001	0.0018	0.4593	0.9402	0.8446

Supplementary File 4 Principal component analysis for the cutoff age

Variable	<20		20-50		≥50	
	Estimate	*P* value	Estimate	*P* value	Estimate	*P* value
SO_2	0.2369	0.2912	0.2347	0.4198	-2.8406	0.0451
NO_2	0.3130	0.1236	0.5869	0.0273	2.6528	0.0506
PM_{10}	0.1223	0.0537	0.0500	0.5480	-0.0163	0.9700
O_3	0.7192	0.0000	0.4973	0.0005	-0.0817	0.9106
$PM_{2.5}$	0.0019	0.9884	0.0859	0.5977	-0.2974	0.7104
temp.av	3.5536	0.0000	3.7688	0.0001	10.6424	0.0072
temp.hi	3.1007	0.0000	3.2072	0.0001	9.0609	0.0115
temp.lo	2.7642	0.0008	3.4234	0.0006	11.0969	0.0055
pres.av	-1.1897	0.0576	-0.6774	0.3932	-4.0442	0.2545
prec	0.0002	0.7300	-0.0001	0.8655	-0.0030	0.4653
humi.av	-1.0184	0.0005	-0.9148	0.0170	-1.1010	0.5858
velo.av	-1.3714	0.7217	6.8841	0.1786	73.3291	0.0060
